# Advances in the Discovery and Engineering of Gene Targets for Carotenoid Biosynthesis in Recombinant Strains

**DOI:** 10.3390/biom13121747

**Published:** 2023-12-05

**Authors:** Buli Su, Ming-Rong Deng, Honghui Zhu

**Affiliations:** Key Laboratory of Agricultural Microbiomics and Precision Application (MARA), Key Laboratory of Agricultural Microbiome (MARA), State Key Laboratory of Applied Microbiology Southern China, Institute of Microbiology, Guangdong Academy of Sciences, Guangzhou 510070, China; bolysu@hotmail.com

**Keywords:** metabolic engineering, gene targets, carotenoid, lycopene, β-carotene, astaxanthin

## Abstract

Carotenoids are naturally occurring pigments that are abundant in the natural world. Due to their excellent antioxidant attributes, carotenoids are widely utilized in various industries, including the food, pharmaceutical, cosmetic industries, and others. Plants, algae, and microorganisms are presently the main sources for acquiring natural carotenoids. However, due to the swift progress in metabolic engineering and synthetic biology, along with the continuous and thorough investigation of carotenoid biosynthetic pathways, recombinant strains have emerged as promising candidates to produce carotenoids. The identification and manipulation of gene targets that influence the accumulation of the desired products is a crucial challenge in the construction and metabolic regulation of recombinant strains. In this review, we provide an overview of the carotenoid biosynthetic pathway, followed by a summary of the methodologies employed in the discovery of gene targets associated with carotenoid production. Furthermore, we focus on discussing the gene targets that have shown potential to enhance carotenoid production. To facilitate future research, we categorize these gene targets based on their capacity to attain elevated levels of carotenoid production.

## 1. Introduction

Carotenoids, such as lycopene, β-carotene, and astaxanthin, are synthesized as hydrocarbons or their oxygenated derivatives by a variety of organisms, including plants, fungi, and bacteria [1]. These pigments have garnered significant interest due to their intriguing properties and, more significantly, their potential advantageous impacts on human health [2]. Most carotenoids exhibit a C40 carbon skeleton featuring a C22 central unit comprising nine conjugated double bonds and four side-chain methyl groups. The presence of an alternating double-bond–single-bond system accounts for the high reactivity of carotenoids [3]. Over the past few years, there has been a significant increase in interest regarding the production of natural carotenoids through microbial fermentation. This heightened interest can be attributed primarily to the growth of specific industries, including agriculture, particularly aquaculture and the poultry industry, as well as the nutritional supplement and food industries. In these sectors, natural carotenoids are utilized as coloring agents for various products, such as cooked sausages, soft drinks, baked goods, and pharmaceuticals, and are also incorporated as additives in cosmetics [4]. According to projections, the global carotenoid market is anticipated to attain a value of USD 2.7 billion by 2027, reflecting a compound annual growth rate (CAGR) of 5.7% during the forecast period spanning from 2022 to 2027, up from USD 2.0 billion in 2022 [5]. Consequently, there has been a significant amount of research carried out on carotenogenic microorganisms like *Haematococcus pluvialis*, *Xanthophyllomyces dendrorhou*, and *Blakeslea trispora*, with the aim of achieving large-scale carotenoid production [6].

At present, the majority of industrially produced carotenoids are synthesized chemically using multistep processes or extracted using solvents from non-microbial sources [7]. However, the intricate structure of most carotenoids renders their chemical synthesis an impractical means of production. In contrast, the microbial production of carotenoids offers an environmentally sustainable alternative to chemical techniques and has the potential to meet the increasing need for natural carotenoids [8]. Carotenoid genes were introduced into non-carotenogenic microbes like *Escherichia coli*, *Saccharomyces cerevisiae*, and *Yarrowia lipolytica*, resulting in the successful production of carotenoids [9]. The manipulation of microorganisms to produce different carotenoids has become easier due to recent progress in metabolic engineering and synthetic biology, offering a potentially more sustainable approach to carotenoid production. These efforts have primarily focused on optimizing native pathways, introducing foreign genes to enhance metabolic flux, and achieving co-factor balance [10].

The identification of various gene targets that demonstrate different mechanisms of action in relation to a desired phenotype is an essential aspect of engineering strains. The successful enhancement of cellular phenotypes has been achieved through the utilization of systematic and combinatorial genetic approaches to identify targets for gene knockout and overexpression. In this review, we summarize the latest advancements in the exploration and manipulation of gene targets involved in carotenoid biosynthesis (such as lycopene, β-carotene, and astaxanthin) in recombinant strains (specifically, *E. coli*, *S. cerevisiae*, and *Y. lipolytica*). We investigate gene targets in the carotenoid synthetic pathway and in other pathways associated with and outside the carotenoid biosynthetic pathway. However, we do not address the systematic manipulation of metabolic pathways for strain engineering. We encourage interested readers to consult a recent review on this topic [11].

## 2. Biosynthesis of Carotenoids

**Figure 1:**
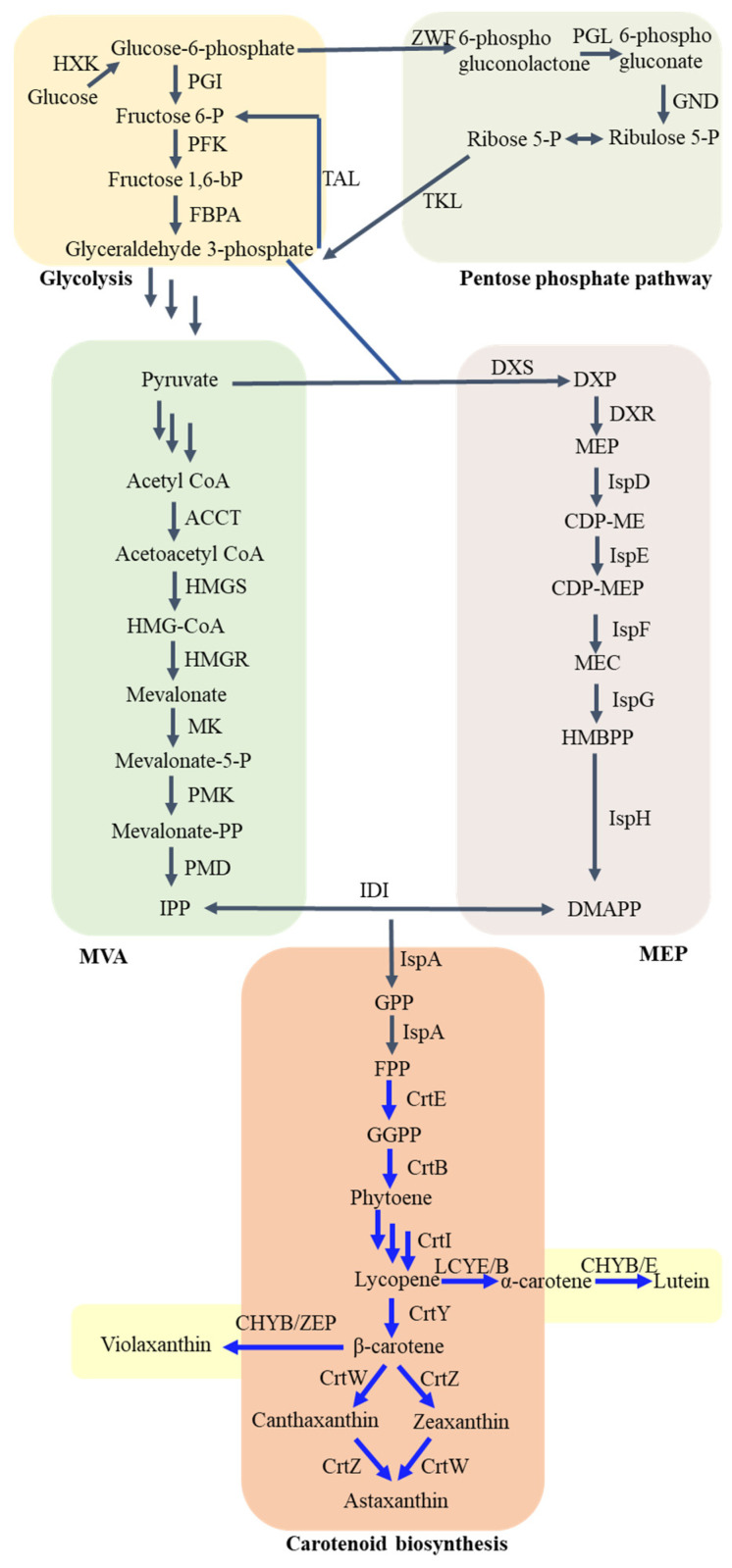
Overview of metabolic pathways for carotenoid biosynthesis. HXK, hexokinase; PGI, phosphoglucose isomerase; PFK, phosphofructokinase; FBPA, fructose-bisphosphate aldolase; ZWF, glucose-6-phosphate dehydrogenase; GND, 6-phosphogluconate dehydrogenase; TKL, transketolase; TAL, transaldolase; DXP, 1-deoxy-*D*-xylulose-5-phosphate; MEP, methylerythritol phosphate; CDP-ME, 4-diphosphocytidyl-*2C*-methyl-d-erythritol; CDP-MEP, 4-diphosphocytidyl-*2C*-methyl-*D*-erythritol-2-phosphate; MEC, *2C*-methyl-*D*-erythritol-2,4-cyclo-diphosphate; HMBPP, 4-hydroxy-3-methyl-2-(*E*)-butenyl-4-diphosphate; HMG-CoA, 3-hydroxy-3-methylglutaryl-CoA; Mevalonate-5-P, mevalonate-5-phosphate; Mevalonate-PP, mevalonate-5-diphosphate; IPP, isopentenyl diphosphate; DMAPP, dimethylallyl diphosphate; GPP, geranyl diphosphate; FPP, farnesyl pyrophosphate; GGPP, geranylgeranyl diphosphate; DXS, DXP synthase; DXR, DXP reductoisomerase; IspD, CDP-ME cytidylyltransferase; IspE, CDP-ME kinase; IspF, MEC synthase; IspG, HMBPP synthase; IspH, HMBPP reductase; ACCT, acetoacetyl-CoA thiolase; HMGS, HMG-CoA synthase; HMGR, HMG-CoA reductase; MK, mevalonate kinase; PMK, mevalonate-5-P kinase; PMD, mevalonate-PP decarboxylase; IDI, isopentenyldiphosphate isomerase; IspA, FPP synthase; CrtE, GGPP synthase; CrtB, phytoene synthase; CrtI, phytoene desaturase; CrtY, lycopene cyclase; CrtW, β-carotene ketolase; CrtZ, β-carotene 3-hydroxylase; LCYB, lycopene β-cyclases; LCYE, lycopene ε-cyclases; CHYB, carotene β-hydroxylase; CHYE, carotene ε-hydroxylase; ZEP, zeaxanthin epoxidase.

Two distinct pathways, namely, the well-established mevalonate (MVA) pathway and the relatively new 2-*C*-methyl-*D*-erythritol 4-phosphate (MEP) pathway, facilitate the production of isopentenyl diphosphate (IPP) and its isomer dimethylallyl diphosphate (DMAPP). These pathways are crucial for synthesizing all carotenoids [12,13]. DXS catalyzes the condensation of G3P and pyruvate to produce DXP, as shown in Figure 1. Following that, DXP reductoisomerase (DXR or IspC) facilitates the transformation of DXP into MEP as part of the MEP pathway. Moreover, a sequential enzymatic pathway that includes multiple enzymes, specifically, CDP-ME cytidylyltransferase (IspD), CDP-ME kinase (IspE), MEC synthase (IspF), HMBPP synthase (IspG), and HMBPP reductase (IspH), is accountable for the gradual transformation of MEP into IPP. This pathway also involves the corresponding intermediates CDP-ME, CDP-MEP, MEC, and HMBPP. Afterwards, the enzyme IDI aids in the conversion of IPP to DMAPP through isomerization [14,15].

The start of the MVA pathway includes the transformation of acetyl coenzyme (acetyl-CoA) into MVA via three consecutive reactions, facilitated by acetoacetyl-CoA thiolase (ACCT), 3-hydroxy-3-methylglutaryl-CoA synthase (HMGS), and HMG-CoA reductase (HMGR). Following this, MVA is further converted into mevalonate-5-phosphate (MVAP) by mevalonate kinase (MK). MVAP is converted into IPP through various pathways. Eukaryotes use a pathway that includes two back-to-back reactions aided by MVAP kinase (PMK) and MVAPP decarboxylase (MDD), while archaea utilize a pathway comprising two reactions catalyzed by MVAP decarboxylase (MPD) and isopentenyl phosphate kinase (IPK) [16,17].

After the combination of IPP and DMAPP, the condensation reaction of these two compounds leads to the creation of geranyl diphosphate (GPP). In *E. coli*, *ispA* encodes FPP synthase, which is responsible for the synthesis of GPP and farnesyl diphosphate (FPP). In contrast, *crtE* encodes GGPP synthase, which catalyzes the formation of geranylgeranyl diphosphate (GGPP). The combination of two GGPP molecules, facilitated by phytoene synthase produced by crtB, results in the creation of colorless C40 phytoene. Additional desaturation of phytoene through the action of phytoene desaturase (encoded by *crtI*) leads to the formation of lycopene, which exhibits a red hue due to the presence of 11 conjugated double bonds. To synthesize cyclic carotenoids, lycopene undergoes cyclisation of either one or both of its end groups. Lycopene β-cyclases (encoded by *crtY*) enable the enzymatic process of lycopene cyclisation, leading to the production of β-carotene. α-carotene contains one ε-ring and one β-ring; thus, the conversion of lycopene to α-carotene requires both lycopene β-cyclases (LCYB) and lycopene ε-cyclases (LCYE). Following that, *crtZ* expression leads to β-hydroxylase synthesis, and *crtW* expression produces ketolase, which transform β-carotene into zeaxanthin and canthaxanthin, respectively. Through combined efforts, these enzymes ultimately convert canthaxanthin into astaxanthin. Furthermore, zeaxanthin epoxidase (ZEP) and carotene β-hydroxylase (CHYB) can convert β-carotene to violaxanthin. Similarly, lutein is generated from α-carotene by CHYB and carotene ε-hydroxylase (CHYE) [18,19].

## 3. Technology for Discovering Novel Gene Targets

Identifying gene targets to enhance a specific phenotype through knockout or overexpression is a challenging endeavor [20,21]. Historically, knowledge-based empirical methods have been employed for the straightforward identification of targets within the MEP, MVA, or local central metabolic pathways that could improve carotenoid production [22,23]. Additionally, random approaches led to the discovery of several regulatory targets using the shotgun method, and co-expression of *appY* with *dxs* produced an eight-fold improvement in lycopene yield [24]. In one study, transposon mutagenesis to identify gene targets that could be deleted to improve the supply of cofactors or precursors resulted in a four-fold increase in lycopene yield following the deletion of *gdhA*, *aceE*, and *yjiD* [25]. In another study, the authors examined the potential genes that influence the overall network properties and cellular phenotype by performing a genome-wide stoichiometric flux balance analysis. A total of seven mutants with single or multiple stoichiometric gene deletions were identified, showing a 37% improvement in lycopene yield in a *gdhA/aceE/fdhF* triple-knockout construct [26]. Another group conducted a multi-dimensional search to identify gene targets, and a 3.7-fold increase in lycopene production was observed by the deletion of *gdhA*/*aceE*/*fdhF* and the overexpression of *yjiD*/*ycgW* [27]. The extensive scope and nature of intricately interconnected cellular networks have significantly impeded the identification of new targets across the entire genome [28]. However, the use of computer-based in silico modeling methods has made it easier to systematically discover new genome-scale targets, ultimately improving the efficiency of industrial strains and boosting the production of various bio-products. Various methods, including the minimization of metabolic adjustment (MOMA), the analysis of flux distribution comparison (FDCA), and flux scanning based on enforced objective flux (FSEOF), have been utilized to forecast potential targets for knockout or up-regulation [29,30,31,32].

**Figure 2:**
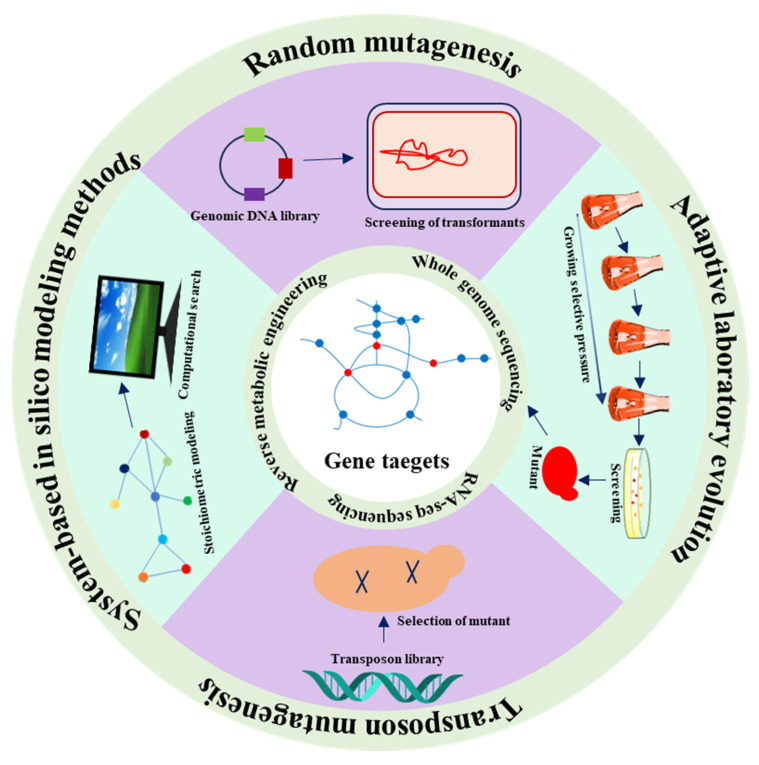
Summary of the methods for the discovery of novel gene targets to enhance carotenoid production. Usually, random mutagenesis, transposon mutagenesis, system-based in silico modeling and adaptive laboratory evolution are conducted to identify favorable mutants. Then, whole-genome sequencing, RNA-seq sequencing, and reverse metabolic engineering are used to revel the gene targets.

The complex structure of cellular metabolic networks and our limited understanding of regulatory information related to the targeted chemicals are obstacles in the advancement of effective biosynthetic systems in microorganisms through conventional metabolic engineering strategies [33]. As a result, metabolic engineering frequently depends on serendipitous findings to augment chemical production [34]. Considering the inherent pigmentation of carotenoids, straightforward, color-centric high-throughput screening techniques have been devised to isolate favorable mutants [35]. Consequently, researchers have developed numerous mutagenic approaches to facilitate carotenoid production. A type of plasma known as atmospheric and room temperature plasma (ARTP) has the potential to induce the production of astaxanthin and allowed the discovery of three specific genes (*CSS1*, *YBR012W-B*, and *DAN4*) linked to astaxanthin biosynthesis. *CSS1* deletion, achieved by ARTP mutagenesis, led to a 75.6% increase in astaxanthin yield [36]. Adaptive laboratory evolution (ALE) proved to be a valuable approach to enhance the phenotype or physiological attributes of strains [34]. Indeed, researchers successfully identified numerous gene targets by using ALE; for example, the overexpression of the class E protein gene *Did2* led to a 2.1-fold increase in β-carotene yields [37,38,39,40]. By employing ALE, our group also identified two novel gene targets, *cho2* and *pfk1*, whose regulation can increase lycopene yield by 3.4 and 5.1 times, respectively [41,42]. Different methods for the discovery of novel gene targets associated with carotenoid synthesis are summarized in Figure 2.

## 4. Gene Targets in the Carotenoid Synthetic Pathway

Microbial cell factory hosts commonly lack the complete set of genes necessary for carotenoid biosynthesis. Consequently, the introduction of heterologous genes is necessary to establish a carotenoid biosynthetic pathway [43]. Wild-type enzymes that have been identified and screened typically exhibit limited activity, affinity, and expression levels in heterologous hosts. To enhance carotenoid production, various strategies involving protein engineering have been devised [44]. It is worth mentioning that the transformation of DMAPP into GGPP through the action of GGPP synthase was recognized as the pivotal step that restricts the rate of carotenoid biosynthesis [45]. To enhance the flow of molecules along this pathway, researchers have employed directed evolution techniques on GGPP synthase to augment carotenoid production [46,47,48].

Similarly, directed evolution was used on a dual-function enzyme consisting of phytoene synthase (crtYB) and lycopene cyclase (crtE) [49] and on the individual enzyme β-carotene ketolase (OBKT) [50], resulting in increased carotenoid production. Furthermore, a mutated form of FPP synthase, which exhibits modified chain-length specificity, could enhance GGPP synthesis and the production of its downstream metabolites [51]. In another study, researchers aimed to enhance lycopene and astaxanthin production in *S. cerevisiae* by a combining metabolic and protein engineering strategies. Specifically, they enhanced the expression of CrtE and produced an engineered CrtI mutant (Y160F&N576S), resulting in a 60% increase in lycopene production [52]. In another study, researchers increased the activity of the rate-limiting enzyme OBKT through protein engineering, obtaining a 34% improvement in astaxanthin production [50]. To address the limitation posed by lycopene as the sole aggregating precursor and its significant impact on carotenoid biosynthesis, researchers employed a structure-guided protein engineering approach. They aimed to mitigate the inhibitory effect of lycopene cyclase through targeted modification. They developed a variant, namely, CarPR(Y27R), that exhibited complete elimination of substrate inhibition while maintaining enzymatic activity [53].

## 5. Gene Targets Involved in the Carotenoid Biosynthetic Pathway

### 5.1. Central Metabolic Pathway

In the MVA pathway, three molecules of acetyl-CoA are required per isoprene unit. Acetyl-CoA also serves as the precursor for lactate, acetate, and ethanol; hence, the removal of these byproducts has the potential to enhance carotenoid production [54]. Furthermore, acetyl-CoA flux into the MVA pathway can be enhanced by reducing the consumption of acetyl-CoA in the tricarboxylic acid (TCA) cycle via the elimination of the gene that encodes a component of 2-oxoglutarate dehydrogenase (SucAB) [55]. In addition, to increase carotenoid production, techniques like redirecting the pentose phosphate (PP) pathway to enhance the availability of acetyl-CoA and converting pyruvate directly to acetyl-CoA have been utilized [56]. Additionally, researchers found that an increase in cytosolic citrate levels enhanced acetyl-CoA synthesis and subsequently promoted lycopene biosynthesis. They overexpressed the AMP deaminase-encoding gene (AMPD) to inhibit the activity of isocitrate dehydrogenase, resulting in an elevated citrate supply and an approximately three-fold increase in lycopene content [57].

**Figure 3:**
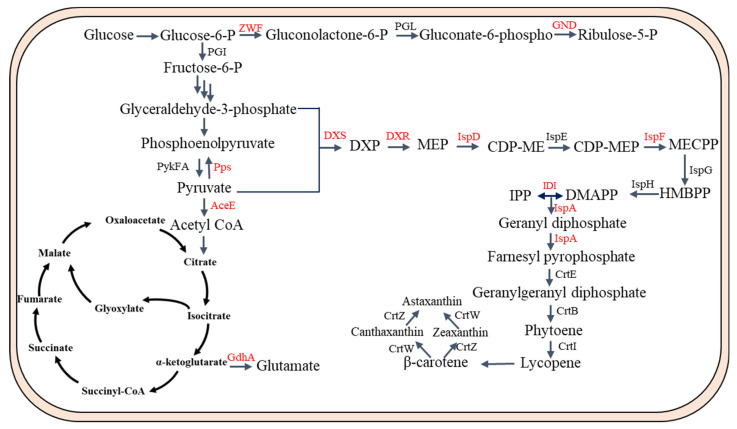
Overview of the gene targets for engineering the regulatory networks to enhance carotenoid production in *E. coli*. Gene targets are labeled in red. Pps, phosphoenolpyruvate synthase; ZWF, glucose-6-phosphate dehydrogenase; GND, 6-phosphogluconate dehydrogenase; PGI, glucosephosphate isomerase; PGL, 6-phosphogluconolactonase; GdhA, glutamate dehydrogenase; PykFA, pyruvate kinases; AceE, pyruvate dehydrogenase.

As shown in Figure 3, many gene targets were identified in the PP pathway and TCA pathway. For instance, both G3P and pyruvate are used in equal amounts as precursors in the MEP pathway. The biosynthesis of carotenoids can be reduced by limiting either of these precursors. The conversion of G3P to pyruvate is the main bottleneck in this pathway, leading to an imbalanced flow towards pyruvate, which is a significant constraint [58]. The authors achieved a 250% increase in lycopene production by overexpressing phosphoenolpyruvate (PEP) synthase (PPS) and diverting the flow from pyruvate to G3P [59]. Furthermore, the Entner–Doudoroff (ED) pathway, known for producing equivalent quantities of G3P and pyruvate, can be utilized and enhanced to attain increased carotenoid synthesis [60]. Moreover, manipulation of the central metabolic pathway, specifically through the knockout of the *zwf* gene, significantly enhanced lycopene production by over 130% [61]. Similarly, deletion of the *zwf* gene increased the β-carotene content in the resultant strain by 32.5% [62]. Conversely, inhibiting the bypass pathway redirected carbon flow towards lycopene synthesis. Researchers knocked out the *aceE* and *gdhA* genes, resulting in an enhanced carbon metabolic flux towards lycopene production [63]. These above studies provide evidence for the potential to manipulate the central metabolic pathway to ensure an adequate supply of precursors for IPP synthesis.

### 5.2. MEP and MVA Pathways

As shown in Figure 3, many gene targets were also identified in the MEP pathway. The combination of pyruvate and G-3-P through the DXS enzyme is widely acknowledged as the step that limits the rate of the MEP pathway [64]. Consequently, by the overexpression of *dxs*, which encodes 1-deoxy-*D*-xylulose-5-phosphate synthase, in *E. coli*, carotenoid production was enhanced 3.5-fold [65]. Furthermore, *dxs* overexpression could greatly enhance β-carotene levels, particularly when optimized for astaxanthin production in *E. coli* [66]. The overabundance of the IspD (MCT) and IspF (MDS) enzymes in the MEP pathway led to a significant boost of 71% in the synthesis of astaxanthin [67]. To boost the production of lycopene, it is crucial to promote the formation of IPP, a product of the MEP/MVA pathway, and guarantee an ample availability of acetyl-CoA or pyruvate. To accomplish this, scientists expressed the foreign MEP pathway genes *dxs* and *idi* simultaneously in *E. coli*. According to their report, there was an increase of 16.5 times in lycopene yield, along with the upregulation of genes in the downstream isoprenoid pathway [68]. In another study, the authors used directed co-evolution of the key enzymes (DXS, DXR, and IDI) of the MEP pathway to enhance lycopene production [69]. IDI is a crucial enzyme in the lycopene biosynthetic pathway and a significant focus of metabolic engineering. To enhance the activity of *S. cerevisiae* IDI, authors employed a directed evolution strategy involving error-prone polymerase chain reaction (PCR). Subsequent fermentation experiments demonstrated that the mutant IDI exhibited a 1.8-fold increase in lycopene production [70].

**Figure 4:**
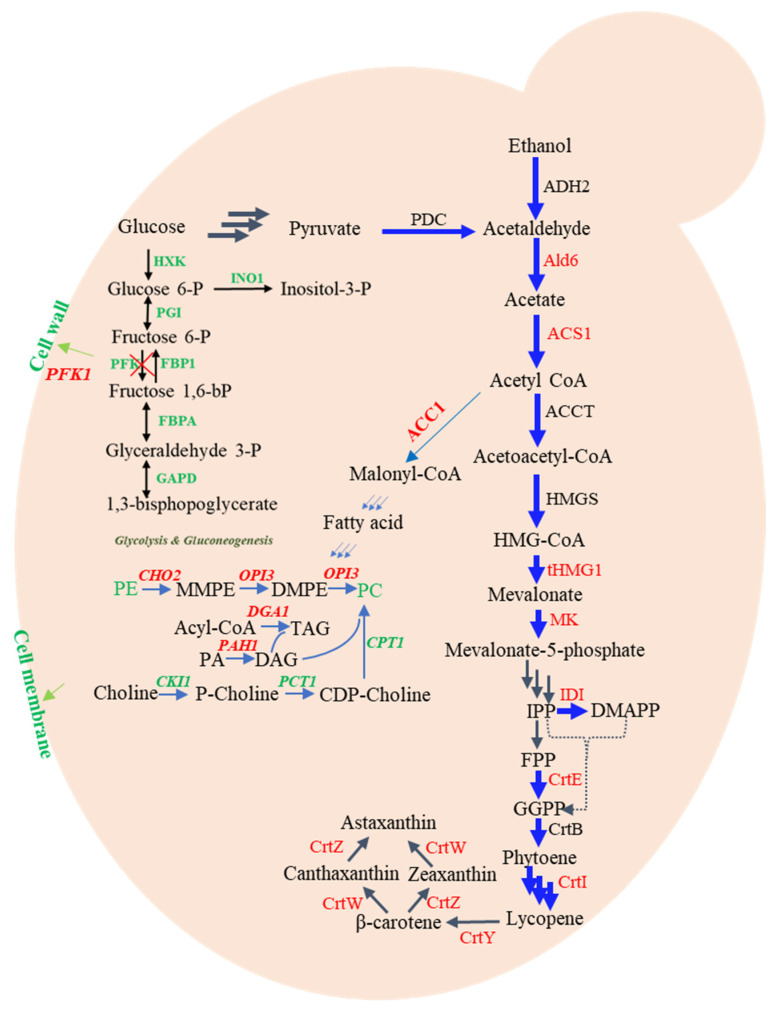
Overview of the gene targets for engineering the regulatory networks to enhance carotenoid production in yeast. Gene targets are labeled in red. PA, phosphatidicacid; DAG, diacylglycerol; TAG, triacylglycerol; PS, phosphatidylserine; PE, phosphatidylethanolamine; MMPE, phosphatidylmonomethylethanolamine; DMPE, phosphatidyldimethylethanolamine; PC, phosphatidylcholine; CHO2, phosphatidylethanolamine N-methyltransferase; OPI3, phosphatidyl-N-methylethanolamine N-methyltransferase; GDA1, Diacylglycerol O-acyltransferase 1; PAH1, phosphatidate phosphatase; CKI1, choline kinase; PCT1, phosphocholine cytidyltransferase; CPT1, choline phosphotransferase.

As shown in Figure 4, many gene targets were identified in the MVA pathway. For example, the addition of the MVA pathway genes *mvak1*, *mvak2*, *mvaD*, and *idi* resulted in the provision of the precursors IPP and DMAPP, leading to a significant > three-fold increase in lycopene production [71]. The MVA bottom pathway facilitates the conversion of MVA into IPP and DMAPP through four enzymatic steps. When researchers overexpressed tHMGR from *X. dendrorhous*, a truncated form of *HMGR* that spans the membrane, *in S. cerevisiae*, there was a 2.2-fold enhancement in β-carotene production [72]. In *Y. lipolytica*, overexpression of the bottleneck genes *HMG1* and *GGS1* led to a remarkable 10.8-fold improvement in lycopene yield [73].

### 5.3. Lipid Pathway

The genes implicated in the formation of lipid droplets have shown promise as viable targets to enhance the production of hydrophobic products [74]. This is because enlarging lipid droplets, which serve as natural repositories for neutral lipids, enable the inclusion of a greater quantity of lipophilic substances [75]. It has been common practice to manipulate the genes involved in the synthesis, size determination, and breakdown of lipid droplets to augment the droplets’ storage capacity and thus enhance carotenoid accumulation [76]. For example, deletion of *FLD1*, a gene known for its ability to control the dimensions of lipid droplets, resulted in a 25% increase in lycopene yield [77]. Similarly, overexpression of *ACC1*, *PHA1*, and *DGA1*, which, respectively, encode acetyl-CoA carboxylase, phosphatide phosphatase (PAP), and diacylglycerol acyltransferase, led to a 22.7% enhancement in β-carotene production [78]. It is worth mentioning that increasing lipid droplets by enhancing lipid synthesis provides a larger storage capacity, although it comes at the cost of redirecting the metabolic flux from the intended pathway. Hence, it is necessary to ensure appropriate spatial control. Furthermore, the recent discovery of *opi3* and *hrd1* as engineering objectives revealed their ability to enhance astaxanthin production by moderately stimulating lipid synthesis rather than excessively upregulating it [79].

*S. cerevisiae*, a yeast that is not oleaginous, has a restricted ability to produce lipophilic substances like β-carotene. Researchers aimed to enhance the accumulation of β-carotene in *S. cerevisiae* by overexpressing the sterol ester synthesis genes *ARE1* and *ARE2*. The results indicated a 1.5-fold increase in β-carotene yield. Additionally, deletion of PAP genes (*PAH1*, *DPP1*, and *LPP1*) led to a two-fold increase in β-carotene yield. The combination of these two strategies resulted in a 2.4-fold improvement in β-carotene production [80]. *Y. lipolytica* possesses lipid bodies that facilitate the storage of β-carotene, which makes it a promising candidate for β-carotene production. By overexpressing *DID2*, β-carotene production was further enhanced by 260% [81].

## 6. Gene Targets Outside the Carotenoid Biosynthetic Pathway

The heterologous biosynthesis of target metabolites in a microbial chassis can be influenced by seemingly unrelated genes. Indeed, several other genes outside the carotenoid pathway affect carotenoid production [82]. As an instance, when *SOD1*, a gene responsible for producing superoxide dismutase, was overexpressed in *S. cerevisiae*, the carotenoid yield increased by 2.6 times [83]. Lycopene yield was also increased 74.6-fold by the simultaneous overexpression of *OLE1*, which encodes delta-9 fatty acid desaturase, and *STB5* [84]. Deletion of the *DAN4* gene, which encodes a cell wall mannoprotein, resulted in a 36.3% increase in astaxanthin production [36]. Similarly, deletion of *HRD1* and overexpression of the transcription factor *Pdr3* led to a 61.61% improvement in astaxanthin yield [85]. Furthermore, deletion of *YPL062W* in *S. cerevisiae* had advantageous effects on carotenoid production by redirecting carbon towards the synthesis of carotenoid precursors, namely, acetyl-CoA and MVA [86].

**Figure 5:**
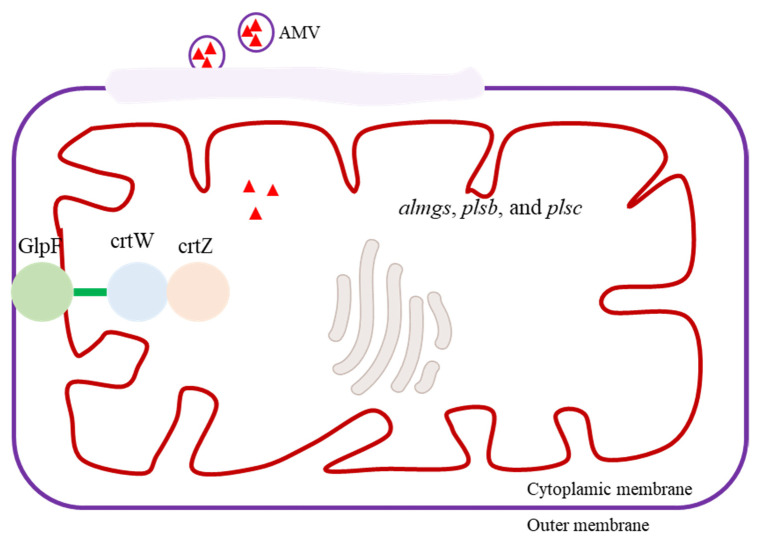
Overview of a membrane engineering strategy to increase carotenoid production. By overexpressing membrane-bending protein (almgs), glycerol-3-phosphateacyltransferase (plsb), and 1-acylglycerol-3-phosphate-acyltransferase (plsc), introducing membrane-bending proteins, and enhancing membrane synthesis pathways, the storage capacity for carotenoid was amplified. The fusion of β-carotene ketolase (crtW) and hydroxylase (crtZ) with the glycerol channel protein GlpF facilitated the localization of the enzymes to the membrane, leading to a remarkable enhancement in carotenoid production. Artificial membrane vesicles (AMVs) were constructed to effectively secrete hydrophobic molecules.

Plants and bacteria primarily store carotenoids in their cell membranes, and it is important to note that an overabundance of carotenoids within cells can harm the host organism [87]. By employing membrane engineering, it is possible to augment the production and storage capacity of carotenoids (Figure 5) [88]. By overexpressing *almgs*, *plsb*, and *plsc*, introducing membrane-bending proteins and enhancing membrane synthesis pathways, the storage capacity for lycopene was augmented 1.32-fold [89]. Additionally, the simultaneous manipulation of membrane morphology and the membrane synthesis pathway exhibited a synergistic impact, resulting in a 2.9-fold enhancement of β-carotene yield [90]. Morphological engineering has also been employed to enhance astaxanthin production [91]. To mitigate the morphological transition in the engineered microorganisms, researchers deleted *CLA4* and *MHY1*, reverting the mycelium to the yeast form and thereby further augmenting β-carotene production by 139% [92]. Due to the intricate interconnections between engineered metabolic pathways and inherent cellular metabolism, as well as their stringent regulation, achieving a balanced metabolic flux necessitated the deletion of exg1 and the overexpression of *POS5*, *ALD6*, and *ACS* (which encodes acetyl-CoA synthetase) [93].

The toxicity of carotenoid products poses a significant obstacle to microbial carotenoid production, and the implementation of transporter-mediated carotenoid secretion presents a promising solution to this issue [94]. Enhancing the capacity of the host organism to store carotenoids while mitigating the toxic effects of these molecules represents a substantial challenge. Following their synthesis, carotenoids are expected to associate with lipid membranes owing to their hydrophobic nature [95]. The localization of enzymes to the membrane can enhance the likelihood of enzyme–substrate interactions, thereby enhancing the overall efficiency of substrate conversion. For example, co-localization of crtZ to the *E. coli* membrane by utilizing the signal peptide derived from the outer membrane protein (OmpF) resulted in a significant 60% increase in astaxanthin production [67]. Likewise, the fusion of crtZ and hydroxylase with the glycerol channel protein GlpF facilitated their localization to the membrane, leading to a remarkable 215% enhancement in astaxanthin production [96]. The manipulation of endogenous plasma membrane ATP-binding cassette (ABC) transporters represents a promising strategy for the efficient efflux of hydrophobic products in *S. cerevisiae* [97]. Furthermore, researchers engineered *E. coli* with a novel transport system utilizing artificial membrane vesicles to effectively secrete hydrophobic molecules, resulting in a notable 61% increase in β-carotene production [98]. To enhance the release rate of lycopene, a highly permeable *E. coli* strain was created by deleting the *lpp*, *nlpI*, *mlaE*, and *tolA* genes [99]. The presence of lipopolysaccharides, which are the primary constituents of the outer membrane in *E. coli*, significantly influences bacterial behavior, particularly outer membrane permeability. Consequently, deletion of the *waaC* and *waaF* genes greatly promoted lycopene production [100]. These examples serve to illustrate the significance of genes beyond the target carotenoid synthetic pathway and emphasize the necessity of acknowledging their crucial roles. The above representative gene targets are listed in Table 1.

## 7. Gene Targets Involved in Regulatory Networks

As previously stated, most research pertaining to carotenoid biosynthesis has concentrated on enhancing the expression of pivotal enzymes that govern the rate of carotenoid production, as well as on eliminating or deactivating alternative pathways that compete for the metabolic flux. Manipulating the regulation of metabolic pathways offers a potential means of reprogramming metabolic genes to enhance the output of the desired products by rectifying any imbalances [101]. To illustrate this, the global regulator cAMP receptor protein (CRP) was subjected to transcriptional engineering through the utilization of error-prone PCR and site-directed mutagenesis, resulting in subtle adjustments to the interconnected metabolic pathways and ultimately leading to improved lycopene production. A mutant strain with an engineered CRP produced approximately 25% more lycopene than the control strain [102]. Additionally, the manipulation of global regulatory proteins such as RpoS, AppY, and Crl also increased lycopene production [103]. It follows that the use of engineered regulators to control gene expression could significantly contribute to the design of metabolic pathways that produce carotenoid.

## 8. Bioprocess Engineering

Aside from the development of an efficient upstream process, separation, purification, and analysis of carotenoids from microbial biomass play a crucial role. To extract carotenoids from the microbial biomass, two essential steps must be undertaken: disruption of the membrane of the microbial cells and extraction of the carotenoids. A variety of techniques for cell disruption can be found in the literature. The choice of the method is highly dependent on the specific microorganism used for carotenoid production and the intended application of the extract [104]. Carotenoids are susceptible to degradation from light, high temperatures, and solvents. Therefore, it is essential to carefully select and implement appropriate steps and procedures to ensure their stability. To achieve this, biomass lyophilization is frequently employed, although it does result in increased time and costs [105]. Additionally, metabolites and cell slurry are collected for the subsequent extraction of carotenoids. Despite the high yields obtained with solvent extraction methods, the chemical compounds commonly used exert harmful effects on human health and the environment, prompting the scholarly community to explore more environmentally friendly alternatives. This objective has been achieved using a variety of techniques, including ultrasound-assisted extraction, microwave-assisted extraction, enzyme-assisted extraction, ionic liquid extraction, and supercritical fluid extraction [106].

## 9. Conclusions and Future Perspectives

This review provides a comprehensive analysis of the current state of microbial carotenoid research, with a focus on the discovery and engineering of gene targets. Given their health benefits and natural origin, biotechnologically produced natural carotenoids are gradually replacing synthetic carotenoids. Hence, the utilization of microorganisms for carotenoid production holds significant promise and offers opportunities within the pharmaceutical and food industries. However, there are several processing challenges in the industrial context, such as the exorbitant expenses associated with the current production and extraction technologies, as well as the reliance on substantial quantities of non-environmentally friendly solvents as extraction agents. We contend that the utilization of integrated upstream and downstream platforms, coupled with environmentally friendly solvents and the advancement of inventive and energy-efficient extraction techniques, will effectively address the existing limitations. Furthermore, advances in scientific research have the potential to enhance the quality and value-added attributes of microbial carotenoids, rendering this field and market highly appealing to numerous biotechnological industries.

## Figures and Tables

**Table 1:** Carotenoid production characteristics by recombinant strains using the representative gene targets discussed in this review.

Types of Gene Target/Organisms	Genetic Modifications	Phenotypic Changes	Reference
In the synthetic pathway			
*S. cerevisiae*	Directed evolution of β-carotene ketolase (BKT)	34% improvement in astaxanthin yield	[50]
*S. cerevisiae*	Engineered CrtI mutant (Y160F&N576S)	60% increase in lycopene yield	[52]
*Y. lipolytica*	Engineered carRP mutant (Y27R)	1441-fold improvement in β-carotene production	[53]
Involved in the synthetic pathway			
*Y. lipolytica*	Overexpressed AMP deaminase-encoding gene *AMPD*	approximately 3-fold increase in lycopene content	[57]
*E. coli*	Deletion of central carbon metabolic gene *zwf*	130% enhancement in lycopene production	[62]
*E. coli*	Directed evolution of isopentenyl diphosphate isomerase (IDI)	2.1-fold increase in lycopene yield	[70]
*S. cerevisia*	Overexpressed the fatty acid desaturase gene OLE1; deletion of the Seipin gene FLD1	25% increase in lycopene yield	[77]
*Y. lipolytica*	Overexpressed the bottleneck genes *HMG1* and *GGS1*	increased the lycopene content 10.8-fold	[73]
*S. cerevisia*	Overexpression of the sterol ester synthesis genes *ARE1* and *ARE2*; deletion of phosphatidate phosphatase (PAP) genes (*PAH1*, *DPP1*, and *LPP1*)	2.4-fold increase in β-carotene yield	[80]
*S. cerevisia*	Deletion of *TGL3*, *TGL4*, and *TGL5*, encoding TAG lipase and SE hydrolase	37% improvement in β-carotene yield	[78]
*S. cerevisiae*	Deletion of *opi3* and *hrd1*	43.5% improvement in astaxanthin yield	[79]
*S. cerevisia*	Deletion of *pfk1*	5.1-fold increase in lycopene yield	[42]
*S. cerevisiae*	Deletion of *cho2*	3.2-fold increase in lycopene yield	[41]
*S. cerevisiae*	Overexpression of *SOD1*	2.6-fold increase in lycopene yield	[83]
Outside the synthetic pathway			
*S. cerevisia*	Deletion of the *HRD1* gene; overexpression of the transcription factor gene *Pdr3*	61.61% higher astaxanthin yield	[85]
*S. cerevisia*	Deletion of *CSS1*	59% improvement in astaxanthin yield	[36]
*S. cerevisia*	Deletion of *YMRCTy1-3*	2.1-fold improvement in astaxanthin yield	[38]
*E. coli*	Deletion of *waaC*	142% higher lycopene yield	[100]
*E. coli*	Overexpression of *Almgs*, *plsb*, *plsc*, and *dgka*	1.32-fold increase in lycopene yield	[89]
*Y. lipolytica*	Deletion of *CLA4* and *MHY1*	139% improvement in β-carotene yield	[92]
*E. coli*	Overexpression of *Almgs*, *Plsb* and *plsc*	2.9-fold increase in β-carotene yield	[90]
*S. cerevisia*	Overexpression of the *DID2* gene	2.1-fold increase in β-carotene yield	[39]
*E. coli*	Deletion of *yadC* and overexpression of *rnb*	32% improvement in astaxanthin yield	[33]
*S. cerevisia*	Deletion of *YPL062W*	146% increase in lycopene yield	[86]
*E. coli*	Localization of crtW to the membrane using the signal peptide of the outer membrane protein OmpF	60% higher astaxanthin production	[67]
*E. coli*	Localization of crtW and crtZ to the cell membrane by the glycerol channel protein GlpF	215% increase in astaxanthin production	[96]
*E. coli*	Deletion of *gdhA*, *eutD*; overexpression of *tpiA*, *ompE*, and *ompN*	174% increase in lycopene titer	[32]
*E. coli*	Deletion of *aceE* and *gdhA*	140.85% increase in lycopene yield	[63]
*Y. lipolytica*	Overexpression of the *DID2* gene	2.6-fold increase in β-carotene yield	[81]
*S. cerevisia*	Deletion of *exg1*; overexpression of *POS5*, *ALD6*, and acetyl-CoA synthetase, *ACS*	55% increase in lycopene production	[93]
*E. coli*	Deletion of *lpp*, *bamB*, *uspE*, and *yggE*	82% higher astaxanthin yield	[91]
*E. coli*	Deletion of *nlpI* and *tolR*; overexpression of *AccABCD* and *PlsBC*	61% increase in β-carotene	[98]
*E. coli*	Deletion of *lpp*, *nlpI*, *mlaE*, and *tolA*	59.34-fold improvement in extracellular lycopene production	[99]
*S. cerevisia*	Overexpression of the ABC transporter gene *Snq2*	5.80-fold higher extracellular β-carotene production	[97]
